# IgA-Targeted *Lactobacillus jensenii* Modulated Gut Barrier and Microbiota in High-Fat Diet-Fed Mice

**DOI:** 10.3389/fmicb.2019.01179

**Published:** 2019-05-24

**Authors:** Jin Sun, Ce Qi, Hualing Zhu, Qin Zhou, Hang Xiao, Guowei Le, Daozhen Chen, Renqiang Yu

**Affiliations:** ^1^State Key Laboratory of Food Science and Technology, Jiangnan University, Wuxi, China; ^2^School of Food Science and Technology, Jiangnan University, Wuxi, China; ^3^The Affiliated Wuxi Maternity and Child Health Care Hospital of Nanjing Medical University, Wuxi, China; ^4^Department of Food Science, University of Massachusetts, Amherst, MA, United States

**Keywords:** high fat diet, immunoglobulin A, *Lactobacillus jensenii*, mucosal barrier, microbiota, hyperlipidemia

## Abstract

IgA-coated *Lactobacillus* live in the mucous layer of the human or mammalian intestine in close proximity to epithelial cells. They act as potential probiotics for functional food development, but their physiological regulation has not yet been studied. We isolated IgA-targeted (*Lactobacillus jensenii* IgA21) and lumen lactic acid bacterial strains (*Pediococcus acidilactici* FS1) from the fecal microbiota of a healthy woman. C57BL/6 mice were fed a normal (CON) or high fat diet (HFD) for 6 weeks and then treated with IgA21 or FS1 for 4 weeks. HFD caused dyslipidemia, mucosal barrier damage, and intestinal microbiota abnormalities. Only IgA21 significantly inhibited dyslipidemia and gut barrier damage. This was related to significant up-regulation of mucin-2, PIgR mRNA expression, and colonic butyrate production (*P* < 0.05 vs. HFD). Unlike IgA21, FS1 caused a more pronounced gut dybiosis than did HFD, and, in particular, it induced a significant decrease in the *Bacteroidales* S24-7 group and an increase in *Desulfovibrionaceae* (*P* < 0.05 vs. CON). In conclusion, IgA-coated and non-coated lactic acid bacteria of gut have been demonstrated to differentially affect the intestinal barrier and serum lipids. This indicates that IgA-bound bacteria possess the potential to more easily interact with the host gut to regulate homeostasis.

## Introduction

The availability of inexpensive, processed fatty foods promotes the spread of obesity worldwide, which is one of the greatest risk factors for the development of metabolic diseases (Ford et al., 2017). Disruption of homeostasis in the gut microbiota is causally linked to the development of host metabolic diseases, including obesity (Rajani and Jia, 2018). Chronic, modest elevations in fasting serum endotoxins can be induced in lean mice that consume a high-fat diet (HFD) (Cani et al., 2007). This results in unfavorable alterations in the gut microbial composition, leading to increased intestinal permeability (Cani et al., 2009). The subsequent translocation of bacteria or their products can result in chronic tissue inflammation, ultimately triggering metabolic diseases and insulin resistance. Therefore, due to its safety and ease of operation, the regulation of intestinal microbiota by food containing probiotics, prebiotics, or polyphenols has attracted wide attention. It has been found that oral administration of specific strains of *Lactobacillus* and *Bifidobacterium* prevented or alleviated metabolic syndrome in animal experiments (Wang et al., 2015). The effect of *Lactobacillus* on HFD-induced obesity, however, is strain-dependent (Qiao et al., 2015), indicating that the interaction between bacteria and their hosts is very complex, highlighting the necessity to understand its mechanism of action to properly screen strains. There is also a need to establish a targeted screening method for the protection of host mucosal barriers.

Secretory immunoglobulin A (sIgA) is an antibody secreted by the mucosal tissue, and it is concentrated in the outer layer of the gut mucus coating specific local microbiota (Rogier et al., 2014). IgA-targeted microbiota derived from healthy individuals may protect host barrier integrity (Kau et al., 2015; Macpherson et al., 2015), and this is related to specific non-pathogenic commensals, including lactobacilli, clostridial species, and *Akkermansia muciniphila* (Bunker et al., 2015; Planer et al., 2016). IgA-coated bacteria often reside in close proximity to the epithelial surface, in contrast to IgA-free bacteria that live within the intestinal lumen (Pabst et al., 2016). IgA-coated bacteria possess a greater chance of acting on intestinal epithelial cells at a closer distance than lumen bacteria due to a more potent uptake of secreted IgA from these bacteria. Conversely, mucous layer renewal speed is very rapid (Johansson, 2012), and the bacteria bound by sIgA will slough off and be excreted with the feces, allowing for their subsequent isolation. Many *Lactobacillus* species are known to possess the most probiotic potential. A number of *Lactobacillus* species can be used in food production, and it is, therefore, of value to isolate IgA-bound *Lactobacillus*. Although *Lactobacillus* is a rare species that is estimated to constitute approximately 0.3% of all bacteria within the human colon (Almonacid et al., 2017), it may be enriched in IgA-targeted microbiota, allowing for easier isolation. This study aimed to isolate IgA-targeted *Lactobacillus* from healthy humans and to provide insights into their potential to protect the mucosal barrier in HFD-fed mice.

## Materials and Methods

### Materials

Pig gastric mucin and fluorescein isothiocyanate-labeled 4.4-kDa dextran (FD4) were both purchased from Sigma-Aldrich (St. Louis, MO, United States). Kits for plasma total cholesterol (TC), triglyceride (TG), low-density lipoprotein cholesterol (LDL-C), and high-density lipoprotein cholesterol (HDL-C), and a kit for catalase (CAT) activity were purchased from Nanjing Jiancheng Bioengineering Institute (Nanjing, Jiangsu, China). ELISA kits for tumor necrosis factor α (TNF-α), interleukin 6 (IL-6), lipopolysaccharide (LPS), and lipopolysaccharide-binding protein (LBP) were purchased from Huijia Biotechnology Co., Ltd. (Xiamen, China). The BCIP/NBT kit for intestinal alkaline phosphatase (IAP) was purchased from Beyotime Biotechnology (Jiangsu, China). The other solvents and reagents were all analytical grade (Sinopharm Chemical Reagent, Shanghai, China).

### Isolation and Identification of IgA-Coated Lactic Acid Bacteria

IgA-coated bacteria were enriched and cultured from fecal samples obtained from 12 healthy females provided by the affiliated Changzhou Maternity and Child Health Care Hospital of Nanjing Medical University according to a protocol (JN20130918) approved by the Internal Ethics Committee of the Institute of Chinese Medical Sciences, Jiangnan University. Written informed consent was obtained from all donors. Participants were women of 20–35 years of age in good health according to self-report who did not smoke or drink alcohol. Exclusion criteria included mastitis, any infectious disease (especially tuberculosis, viral hepatitis, and human immunodeficiency virus infection), cardiovascular disease, metabolic disease (such as diabetes), mental health disorders, cancer or other malignant or degenerative diseases, inability to answer questions, and current participation in any other study related to nutrition or drug intervention. The basic physical characteristics and blood lipid profiles of the patients were within normal range (Supplementary Table S1). The composition of cultured microbiota was analyzed by 16S rRNA gene amplicon sequencing.

IgA-coated bacteria were collected from feces using a magnetic bead-based enrichment. Briefly, feces suspended at 20% in pre-reduced phosphate-buffered saline (PBS) containing 0.5% Tween 20 (PBST) and protease inhibitors (1 mg/mL leupeptin, 1.6 mg/mL aprotinin, Sigma-Aldrich, St. Louis, MO, United States) were homogenized and centrifuged at 400 ×*g* to remove large debris. The supernatant was centrifuged at 8,000 ×*g* to pellet bacteria and washed with PBST for three times. The bacterial pellet was resuspended in pre-reduced PBS supplemented with 0.25% bovine serum albumin (BSA), 5% goat serum, and biotinylated goat anti-human IgA. After washing, biotin was linked to streptavidin-coated magnetic beads. IgA-coated bacteria were separated from the suspension with the aid of a magnet. Collected bacteria were washed three times with BPST and were cultured in De Man Rogosa Sharpe (MRS) broth and gut microbiota medium (GMM) (Goodman et al., 2011). The above procedure was performed in an anaerobic glove cabinet (DROID Instruments and Equipment Co., Ltd., Shanghai, China). Lactic acid bacteria were isolated using MRS agar from the IgA-coated and IgA-free portion of the samples exhibiting the highest abundance of *Lactobacillus* as revealed by 16S rRNA gene amplicon sequencing. Cultures obtained from the more dilute samples were spread onto *Lactobacillus* anaerobic MRS plates with *lactobacillus* vancomycin and bromocresol green agar (LAMVAB). Single colonies randomly selected from LAMVAB were purified by streaking out twice on MRS agar under anaerobic conditions at 37°C. Purity of the isolates was confirmed by repeated streaking and sub-culturing in fresh MRS agar followed by microscopic examination. Bacterial universal primers 27F and 1492R (Supplementary Table S2) were used to amplify the 16S rRNA from genomic DNA. 16S rRNA sequences were blasted at the EzBioCloud web site for bacterial identification. All isolated strains were further typed by amplified fragment length polymorphism (AFLP).

### AFLP Typing

For discrimination of the strains, the AFLP analysis method was performed using chromosomal DNA as described previously (Watanabe et al., 2009). Briefly, total DNA was digested with EcoRI and MseI restriction enzymes, and the DNA fragments were ligated to double-stranded restriction site-specific adaptors, specifically EcoRI-adaptors and MseI-adaptors (Supplementary Table S1). For the pre-selective and selective PCR amplification, primers EcoR1-core/Mse1-core and EcoRI-A/MseI-CA were used, respectively. The 5′ ends of EcoRI primers were labeled with 6-carboxy-fluorescine (FAM). PCR products were analyzed on an ABI PRISM 3130xl Genetic Analyzer (Applied Biosystems), and the AFLP patterns were analyzed and extracted with GeneMapper software v4.0 (Applied Biosystems). Peak height thresholds were set at 200. Bands of the same size in different individuals were assumed to be homologous and to represent the same allele. Bands of different sizes were treated as independent loci, and data were exported in a binary format with ‘1’ representing the presence of a band/peak and ‘0’ representing its absence. Data were analyzed using NTSYS-pc software (Exeter Software, Biostatistics, Inc., NY, United States) version 2.1. The similarity coefficient was determined using the similarity program for qualitative data (SIMQUAL) by incorporating the Dice similarity coefficient. Cluster analysis was performed to construct a tree plot using the unweighted pair-group method with arithmetic averages (UPGMA) in the SAHN program of the NTSYS-pc software.

### Mucus Adhesion and Cell Surface Hydrophobicity of lgA-Coated and IgA-Free Lactic Acid Bacteria

Wells of microtiter plates were coated with pig gastric mucin or BSA (control). Bacteria were suspended in PBS containing 0.5% tween 20 (PBST) to an OD_600_ of 0.5. Bacterial suspensions (100 μL) were added to each well and incubated overnight at 4°C. The wells were washed with PBST. The buffer was poured off, and after the wells dried, bound bacteria were stained with safranin., The absorbance at OD_492_ was then measured in an enzyme-linked immunosorbent assay (ELISA) plate reader. All measurements were performed in triplicate.

Bacterial surface hydrophobicity was measured using the bacterial adhesion to hydrocarbon (BATH) assay as previously described (Rosenberg, 1984). Bacterial cultures were collected at stationary phase and were pelleted by centrifugation. The pellet was washed twice and suspended in KH_2_PO_4_ (0.01 mmol/L, pH 7.0) to an OD_600_ of 0.5 ± 0.05 (A_0_). A volume of 0.15 mL of hexadecane was added to the 4-mL cell suspension, and the mixture was vortexed for 2 min. Samples were stored for 30 min to let phases separate. The absorbance (OD_600_) of the aqueous phase (A) was again determined. Results were expressed as percentage attachment to hexadecane = (1 - A/A_0_)/100.

### Animal Experiments

The animal experiments were performed according to the National Guidelines for Experimental Animal Welfare (MOST of PR China, 2006), and the Jiangnan University Animal Ethics Committee approved all experiments under protocol number 128/16. Four-week-old male C57BL/6 mice were fed *ad libitum* with chow diet or HFD (45% energy from fat, Supplementary Table S3) for 10 weeks. Control mice fed with chow diet (CON) and then gavaged daily with 0.1 mL of sterile PBS. Mice in the experimental groups were fed with HFD for 6 weeks and then gavaged daily with 0.1 mL of sterile PBS (HFD), 10^9^ CFU/mL of *Lactobacillus jensenii* IgA21 (HFD + J), or *Pediococcus acidilactici* FS1 (HFD + P) in PBS, for 4 weeks. Mice were allowed free access to food and water and maintained under a 12-h light-dark cycle at 24°C and constant humidity in soundproof cages. Body weight and water intake were measured every week during the 10 weeks of diet. Food intake was monitored from the first week of treatment. After 10 weeks of treatment, a Comprehensive Laboratory Animal Monitoring System (CLAMS) (Columbus Instruments, Columbus, OH, United States) was used to determine the respiratory exchange ratio (RER). Ambulatory locomotor activity was measured by consecutive beam breaks in adjacent beams. Heat production was calculated by multiplying the calorific value (CV) (3.815 + 1.232 × RER) by the observed VO_2_ (Heat = CV × VO_2_). Mice were sacrificed by cervical dislocation, and a blood sample of ∼100–200 μL was obtained by cardiac puncture. The liver and white adipose tissue were then removed, cleared of blood, and transferred to pre-chilled Eppendorf tubes on ice for weighing. Tissue samples were weighed and fixed in 4% formalin solution or stored at -80°C for further experiments.

A portion of the inguinal white adipose tissue (WAT) and liver was immediately fixed using 4% neutral buffered formalin for 3 days. Tissues were dehydrated and embedded into paraffin for preparation tissue slice (6 μm) and haematoxylin and eosin (H&E) staining. Other samples were stored at -80°C for further analysis.

### Determination of Intestinal Permeability

Mice were fasted for 4.5 h and then gavaged with 100 μL of 22 μg/μL FD4 at 13:00. Serum was obtained at 14:00 by decapitation. The serum FD4 concentration was calculated by comparing samples to serial dilutions of known standards using a Synergy HT fluorometer (BioTek, Winooski, VT, United States) with excitation at 485 nm and emission at 530 nm. A gain of 50 was used for all experiments.

### Tissue and Blood Collection and Plasma Analysis

TC, TG, LDL-C, and HDL-C of serum were examined using the corresponding enzymatic colorimetric assay kits according to the manufacturer’s instructions. Blood glucose was measured using a glucometer (Accu-Check; Roche Diagnostics, Madrid, Spain). TNF-α, IL-6, LPS, and LBP were assayed by ELISA kit according to the manufacturer’s instructions. IAP activity was determined using the BCIP/NBT kit.

### Analysis of Community Structure by 16rRNA Gene Amplicon Sequencing

The community structure of colonic microbiota was analyzed by 16S rRNA gene amplicon sequencing. Total genomic DNA was extracted from two BAC samples using a soil DNA extraction kit (Mo Bio Laboratories, Carlsbad, CA, United States) following the manufacturer’s protocol. PCR amplicon libraries were constructed for Illumina MiSeq sequencing using bacterial primers targeting the V3-4 hypervariable regions of the 16S rRNA genes (Goodman et al., 2011). Each 20 μL reaction mixture included 5 × FastPfu Buffer, 2.5 mM dNTPs, FastPfu Polymerase, 5 μM of each primer, and 10 ng of template DNA. The PCR profile was set as follows: 95°C for 5 min and 27 cycles at 95°C for 30 s, 55°C for 30 s, and 72°C for 45 s, with a final extension at 72°C for 10 min. Reads from the original DNA fragments were merged using FLASH^1^ (Magoč and Salzberg, 2011), and quality filtering was performed according to the literature (Caporaso et al., 2010). Sequencing data were processed using the Quantitative Insights Into Microbial Ecology (QIIME) pipeline. Operational Taxonomic Units (OTUs) were selected using a *de novo* OTU selection protocol with a 97% similarity threshold. The taxonomic identities of the bacterial sequences were determined using the RPD classifier (Wang et al., 2007). The microbial diversity was analyzed using QIIME software15 with Python scripts. Taxonomy assignment of OTUs was performed by comparing sequences to the Greengenes database (gg_13_5_otus). The raw data were uploaded to the SRA database, and the BioProject ID is PRJNA523678.

### Statistical Analyses

The statistical significance of the comparisons between multiple groups was performed by ANOVA followed by Tukey’s or Duncan *post hoc* tests with normal distributions. Non-parametric data were analyzed with the Kruskal-Wallis H test. Data from microbiota sequencing were analyzed online by MicrobiomeAnalyst (Dhariwal et al., 2017) to calculate alpha diversity scores and Bray–Curtis distances. The UniFrac metric was used to determine the dissimilarity between any pair of bacterial communities. The similarity relationship, assessed using the UniFrac metric, was presented in PCoA (Principal Coordinate Analysis) plots. To identify differences in microbial communities between the three groups, analysis of similarities (ANOSIM) was performed between each pair. Differences in a specific genus were analyzed using the EdgeR package provided by MicrobiomeAnalyst that uses shrinkage estimators, fold change values, and controls false discovery rate by calculating adjusted *P*-Values.

## Results

### Community Structure of Cultivable IgA-Coated Microbiota

**FIGURE 1:**
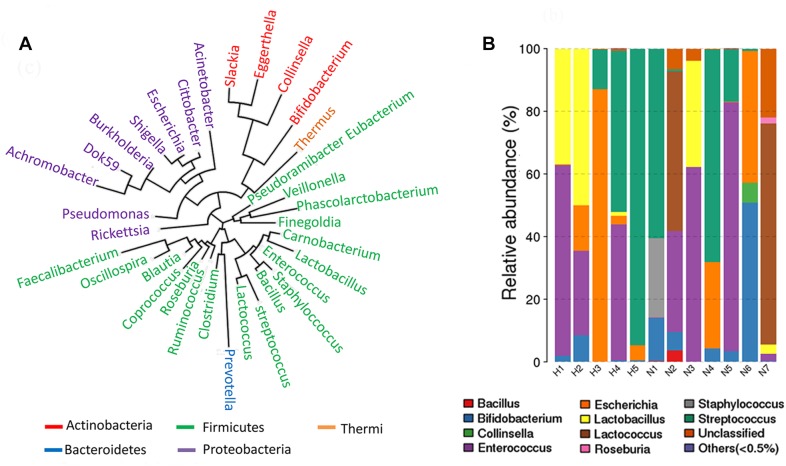
Community structure of IgA-coated bacteria cultured in gut microbiota medium (GMM) and De Man Rogosa Sharpe (MRS) for samples from healthy female donors. **(A)** A phylogenetic tree was constructed based on MRS and GMM cultured IgA-coated microbiota from 12 healthy female donors. **(B)** Genus composition of MRS and GMM cultured IgA-coated microbiota from 12 healthy female donors.

IgA-coated microbiota was separated using a magnetic sorting method from one feces sample provided by a healthy female. To recover IgA-coated bacteria with the best diversity, enrichment of bacteria was performed using MRS medium and gut microbiota medium (GMM). Typical subgroup bacteria were cultured with MRS medium and GMM according to Venn diagrams comparing the operational taxonomic unit (OTU) memberships (Supplementary Figure S1a). Seven rare OTUs were also enriched. The composition of IgA-free microbiota was similar to that of total feces microbiota. GMM-enriched IgA-coated and free microbiota both similar compositions (Supplementary Figure S1b). The MRS-enriched IgA-coated microbiota, however, was clearly different, and it was composed of *Bifidobacterium*, *Escherichia*, and *Lactobacillus*. A genus phylogenetic tree was constructed from sequencing data of MRS- and GMM-cultured IgA-coated microbiota obtained from 12 healthy female donors. The IgA-coated microbiota primarily consisted of Firmicutes, Proteobacteria, and Actinobacteria (Figure 1A). The most frequently occurring genera were *Enterococcus* (6/12), *Streptococcus* (6/12), *Bifidobacterium* (7/12), and *Lactobacillus* (5/12) (Figure 1B).

### Isolation and Identification of IgA-Coated and Lumen Lactic Acid Bacteria

**FIGURE 2:**
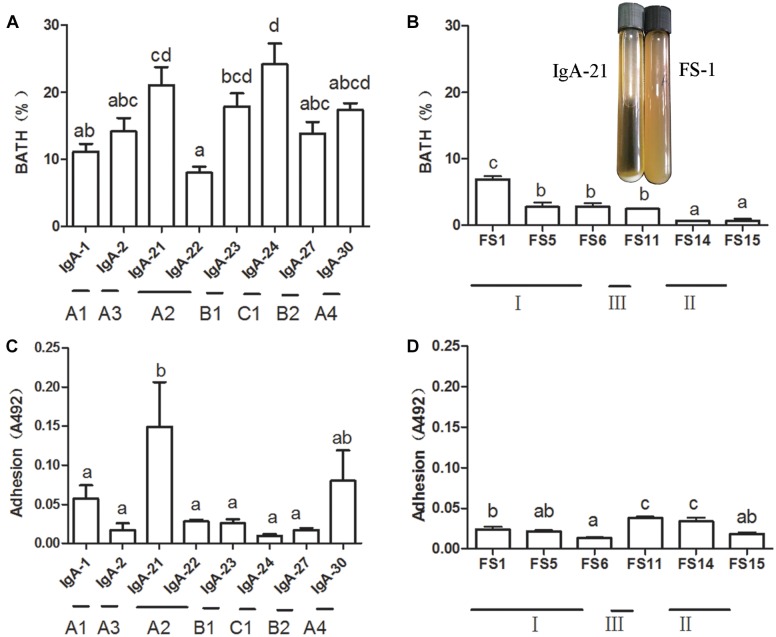
Mucus adhesion and cell surface hydrophobicity of *Lactobacillus jensenii* of different amplified fragment length polymorphism (AFLP) patterns **(A,C)** and *Pediococcus acidilactici* FS1 **(B,D)** and growth state in liquid medium of *L. jensenii* IgA-21 and *P. acidilactici* FS-1. Data are presented as mean ± SD (*n* = 6). Columns with different letters represent significant difference at *P* < 0.05 between different strains. Comparisons were made with one-way ANOVA followed by Tukey’s multicomparison test. BATH: bacterial adhesion to hydrocarbon.

An enriched sample of IgA-coated bacteria containing a high *Lactobacillus* level was chosen as a source from which to isolate lactic acid bacteria. A portion of the larger milky white opaque colonies on MRS agar that exhibited irregular edges were identified as *Weissella confusa*. Other colonies were transparent and smaller with regular edges. All strains isolated from IgA-positive microbiota were identified as *L. jensenii* (Supplementary Table S4). All strains isolated from IgA-free (lumen) microbiota were identified as *Pediococcus acidilactici*, which is similar to *Pediococcus lolii* NGRI 0510QT (Wieme et al., 2012). *L. jensenii* and *P. acidilactici* were included in 3 AFLP patterns (Supplementary Figure S3 and Supplementary Table S4). We then measured the cell surface hydrophobicity of these bacteria, as this property is important for bacteria adhesion within the gut (Krasowska and Sigler, 2014). The typical strains of *L. jensenii* present in different AFLP patterns exhibited a higher cell surface hydrophobicity than that of *Pediococcus acidilactici* (Figure 2A,B). It has been suggested that the aggregation and adhesion characteristics of beneficial bacteria contribute to intestinal colonization, thereby enhancing the mucosal barrier to resist pathogenic bacteria infection (Tareb et al., 2013). *L. jensenii* isolated in our study displayed typical self-aggregation, while *P. acidilactici* did not. At the same time, *L. jensenii* exhibited strain-dependent mucus adhesion, with IgA21 exhibiting the highest adhesion (Figure 2C). All *P. acidilactici* strains, however, showed very weak mucus adhesion *in vitro* (Figure 2D). Additionally, IgA21 and *P. acidilactici* FS1 are typical slow-growing and fast-growing bacteria, respectively. (Supplementary Figure S2).

### General Features of HFD-Fed Mice

From the 4th week of the animal experiments, the weight of the mice in the group fed with HFD was significantly higher from that of the normal diet fed group (*P* < 0.01) (Supplementary Figure S4a). Notably, IgA21 and FS1 exhibited no significant effect on weight gain compared to that observed with HFD fed control mice (Supplementary Figure S4b). The liver of mice in the HFD + P group exhibited slight fat infiltration and white adipose tissue cell enlargement, but the liver structure and adipocytes of mice treated with J were normal (Supplementary Figures S4c,d).

**FIGURE 3:**
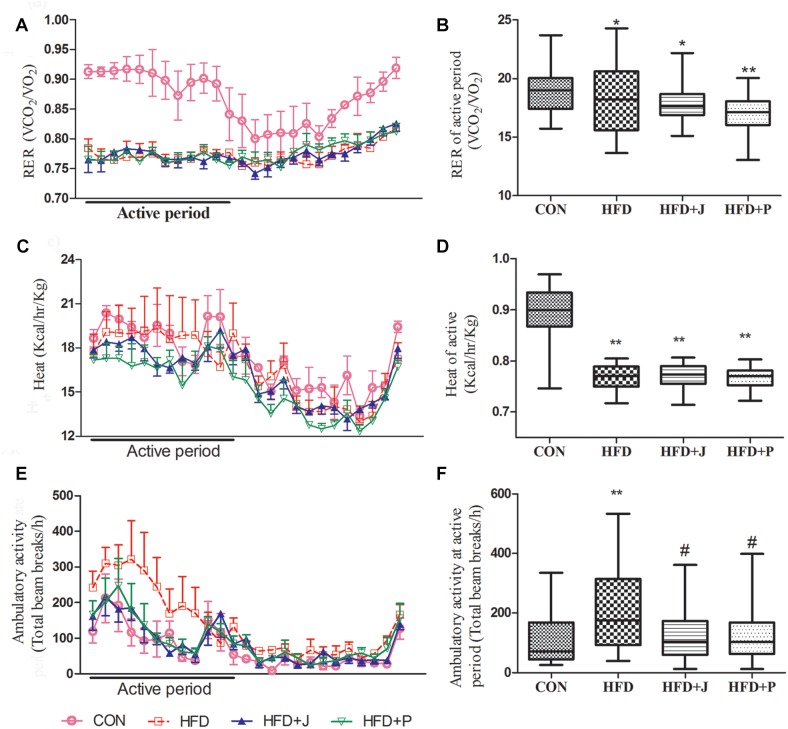
Effects of *Lactobacillus jensenii* IgA-21 and *Pediococcus acidilactici* FS-1 on the respiratory exchange ratio (RER) **(A,B)**, heat **(C,D)**, and ambulatory activity **(E,F)** in high-fat diet (HFD) mice. Bar graphs represent median range values during the active cycle over 48 h. CON, control group; HFD, high-fat diet group; HFD + J, high-fat diet group treated with *L. jensenii* IgA-21; HFD + P, high-fat diet group treated with *P. acidilactici* FS1. RER, respiratory exchange ratio. Data of **A**, **C** and **E** are presented as mean ± SD (*n* = 8). ^∗^*P* < 0.05 (HFD versus CON); ^∗∗^*P* < 0.01 (HFD versus CON); #*P* < 0.05 (bacterial treatment versus HFD). Comparisons were made with Kruskal-Wallis ANOVAs and the Bonferroni *post hoc* method to arrive at adjusted *P*-values.

During the 24-h monitoring period in CLAMS, the RER of mice in the HFD group ranged from 0.72 to 0.81 (Figure 3A,B), and heat production was significantly lower than that of mice in CON group (*P* < 0.01, Figure 3C,D). HFD induced a significant increase in ambulatory activity (*P* < 0.01), which was inhibited by both IgA21- and FS1-treatment (*P* < 0.05) (Figure 3E,F).

The serum T-CHOL (*P* < 0.05), TG (*P* < 0.01), and LDL-C (*P* < 0.01) of mice fed with HFD were all increased significantly. Only IgA21 treatment significantly inhibited the increase in TG (*P* < 0.01) (Table 1).

**Table 1:** Effect of *Lactobacillus jensenii* IgA-21 and *Pediococcus acidilactici* FS-1 on serum lipid profile and glucose in HFD feeding mice.

	CON	HFD	HFD+J	HFD+P
LDL-C (mmol/L)	1.30 ± 0.55	2.67 ± 0.27^∗∗^	1.89 ± 0.56	2.25 ± 0.18^∗^
HDL-C (mmol/L)	3.19 ± 0.59	2.49 ± 0.51	3.82 ± 0.60^#^	2.80 ± 0.97
Glucose	7.25 ± 2.11	9.06 ± 0.78	7.82 ± 2.87	7.88 ± 1.82
TG	1.17 ± 0.16	2.41 ± 0.03^∗∗^	1.47 ± 0.38^##^	1.70 ± 0.44
T-CHOL	3.72 ± 0.69	5.38 ± 0.52^∗^	4.56 ± 0.49	4.21 ± 0.0.98


*Data are mean ± SD (*n* = 8 for each group), ^∗^*P* < 0.05, ^∗∗^*P* < 0.01 compared with CON; ^#^*P* < 0.05, *^##^P* < 0.01 compared with HFD; Comparisons were made with Kruskal-Wallis ANOVAs and the Bonferroni *post hoc* method to arrive at adjusted *p*-values. CON, control group; HFD, high-fat diet group; HFD + J, high-fat diet group treated with *L. jensenii* IgA-21; HFD + P, high-fat diet group treated with *P. acidilactici* FS1. LDL-C, Low Density Lipoprotein Cholesterol; HDL-C, High Density Lipoprotein Cholesterol, TG, triglyceride; T-CHOL total cholesterol.*

### Effects of IgA21 on Endotoxemia and Systemic Inflammation in Mice Fed With HFD

**FIGURE 4:**
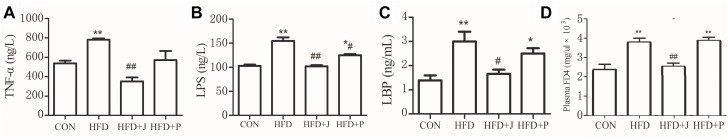
Effects of *Lactobacillus jensenii* IgA-21 and *Pediococcus acidilactici* FS-1 on chronic inflammation (serum TNF-α, **A**), endotoxemia **(B)**, LBP **(C)** and mucosal barrier (FD4) **(D)** in high fat diet fed mice. All data are presented as mean ± SD (*n* = 8). ^∗^*P* < 0.05, ^∗∗^*P* < 0.01 (HFD versus CON); #*p* < 0.05, ##*p* < 0.01 (bacterial treatment versus HFD). Comparisons were made with one-way ANOVA followed by Tukey’s multicomparison test. CON, control group; HFD, high-fat diet group; HFD + J, high-fat diet group treated with *Lactobacillus jensenii* IgA-21; HFD + P, high-fat diet group treated with *Pediococcus acidilactici* FS1. FD4, Fluorescein isothiocyanate-labeled 4.4-kDa dextran; LBP, lipopolysaccharide-binding protein; LPS, lipopolysaccharide; TNF-α, tumor necrosis factor α.

For marker of endotoxemia, serum endotoxin and LBP were measured using TNF-α and IL-6 levels as indices of systemic inflammation (Figure 4). HFD feeding increased circulating concentrations of serum endotoxin (*P* < 0.01), LBP (*P* < 0.05), and TNF-α (*P* = 0.058) (Figure 4). Only IgA21 significantly inhibited HFD-induced endotoxemia and significantly reduced serum LBP and TNF-a (*P* < 0.04 vs. HFD) (Figure 4D).

### Effects of IgA21 on Mucosal Barrier-Related Marker in HFD-Fed Mice

**FIGURE 5:**
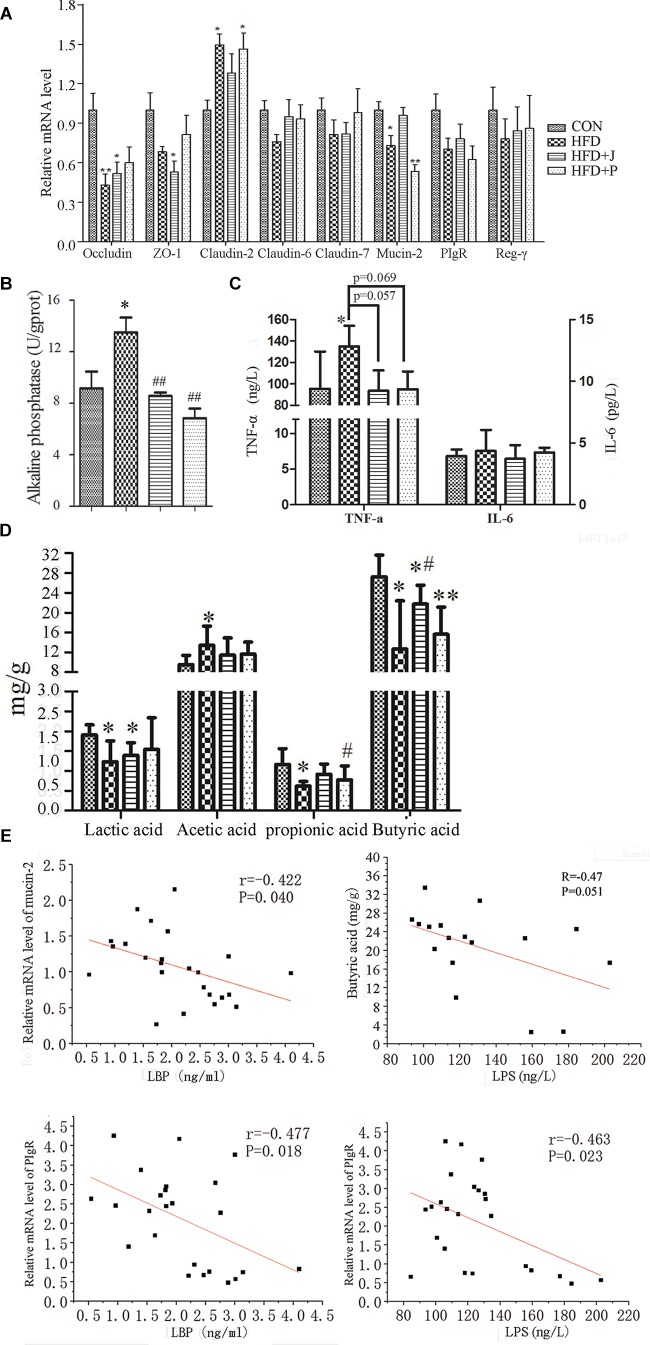
Effects of *Lactobacillus jensenii* IgA-21 and *Pediococcus acidilactici* FS-1 on mucosal barrier related markers. **(A)** mRNA levels of tight junction-related proteins, mucin-2, polymeric immunoglobulin receptor (PIgR), and Reg-γ of ileum measured using quantitative reverse transcription polymerase chain reaction (RT-qPCR). Gene expression was normalized to β-actin. **(B)** Alkaline phosphatase activity of ileum. **(C)** Tumor necrosis factor α (TNF-α) and interleukin 6 (IL-6) production in colon determined by ELISA. **(D,E)** Correlation between serum lipopolysaccharide-binding protein (LBP) and mucin-2 and PIgR mRNA expression; between serum lipopolysaccharide (LPS) and fecal butyric acid and mucin-2 mRNA expression. CON, control group; HFD, high-fat diet group; HFD + J, high-fat diet group treated with *Lactobacillus jensenii* IgA-21; HFD + P, high-fat diet group treated with *Pediococcus acidilactici* FS-1. All data are presented as mean ± SD (*n* = 8). ^∗^*P* < 0.05, ^∗∗^*P* < 0.01 (HFD versus CON); #*P* < 0.05, ##*P* < 0.01 (bacterial treatment versus HFD). Comparisons were made with one-way ANOVA followed by Tukey’s multicomparison test.

To confirm if reversed metabolic endotoxemia by IgA21 was related to improved gut integrity, we measured *in vivo* intestinal permeability to FD4, a selective marker of paracellular permeability (Laukoetter et al., 2006). Serum FD4 concentrations were significantly increased in HFD mice 4 h after gavage, and these concentrations were significantly decreased after IgA21 treatment (*P* < 0.05). To identify candidates targeted by IgA21 in modulation of gut barrier, we initially studied colonic mRNA expression of several tight junction proteins, mucin-2, polymeric immunoglobulin receptor (PIgR), and mRNA levels of RegIIIγ antimicrobial peptides that play an important role in the establishment and maintenance of the mucosal barriers (Okumura and Takeda, 2017). IgA21 significantly prevented HFD-induced down-regulation of Mucin-2 and PIgR mRNA expression (Figure 5A). FS1 could not inhibit the down-regulation of these two genes, and it even led to a more significant decrease of mucin 2 expression compared to CON levels (*P* < 0.05) (Figure 5A). We further determined the activity of IAP enzyme, which is capable of detoxifying LPS (Bates et al., 2007). IAP activity in the colons of mice fed with HFD decreased significantly (*P* < 0.05) (Figure 5B), and this was accompanied by a significant increase in TNF-a production (*P* < 0.05, Figure 5C). Both bacterial treatments significantly inhibited the decrease in IAP activity (*P* < 0.01 vs. HFD), while IgA21 treatment also inhibited the excessive production of TNF-a (*P* < 0.05). Finally, we studied the effects of two bacteria on the production of short-chain fatty acids within the colon, where butyric acid is reported to play a role in maintaining mucosal barrier (Peng et al., 2009). The production of lactic acid, acetic acid, propionic acid, and butyric acid in colon lumens decreased significantly after HFD feeding (*P* < 0.05 vs. CON) (Figure 5D). IgA21 inhibited the decrease in butyric acid, while FS1 only alleviated the decrease in propionic acid production (*P* < 0.05 vs. HFD). Additionally, colonic CAT activity was also sensitive to HFD and was significantly decreased (*P* < 0.05), and this was reversed after IgA21 treatment (*P* < 0.05 vs. HFD, Supplementary Figure S5).

A significant negative correlation was observed between mucin-2 mRNA expression and serum LBP concentration (*r* = -0.42, *P* = 0.04) (Figure 5E). Colon butyric acid and serum endotoxin concentration exhibited a negative correlation trend (*r* = -0.47, *P* = 0.051). PIgR mRNA expression was negatively correlated with LPS (*r* = -0.463, *P* = 0.02) and LBP (*r* = -0.478, *P* = 0.02). This further indicates that mucin-2, the PIgR gene, and butyric acid may mediate the protective effect of IgA21 on the mucosal barrier.

### Differential Modulation of Gut Microbiota Structure by IgA21 and FS1

**FIGURE 6:**
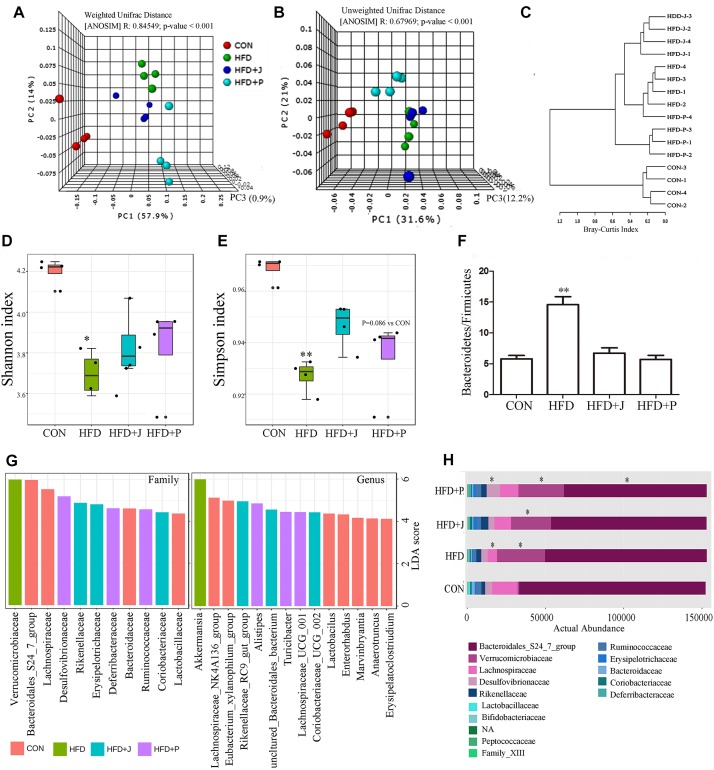
Effect of *Lactobacillus jensenii* IgA-21 and *Pediococcus acidilactici* FS1 treatment on gut microbiota community structure after high-fat-diet feeding. **(A)** Microbial communities clustered using PCoA of the weighted UniFrac matrix. **(B)** Microbial communities clustered using PCoA of the unweighted UniFrac analysis. **(C)** Dendrogram of Hierarchical clustering analysis with distance measure based on Bray-Curtis index. **(D)** Evaluation of α Diversity by Shannan Index. **(E)** Evaluation of α Diversity by Simpson Index. **(F)** Ratio of Bacteroidetes to Firmicutes **(G)** Linear discriminant analysis (LDA) effect size (LEfSe) taxonomic cladogram based on 16S rRNA gene sequences on family and genus level (only LDA more 4 were listed). **(H)** Comparison of bacterial at family level. ^∗^*P* < 0.05, ^∗∗^*P* < 0.01 compare with CON; Comparisons were made with Kruskal-Wallis ANOVAs and the Bonferroni *post hoc* method to arrive at adjusted *p*-values. CON, control group; HFD, high-fat diet group; HFD + J, high-fat diet group treated with *L. jensenii* IgA-21; HFD + P, high-fat diet group treated with *P. acidilactici* FS1.

We studied the gut microbiota of four groups of mice by DNA sequencing. PCR was used to amplify the V3-V4 hypervariable regions of the 16S rRNA gene, and products were then sequenced after multiplexing. yielding yield of 621,991 high quality sequences was obtained. Sequences were clustered into operational taxonomic units (OTUs) with 97% pairwise sequence identity, and they were then assigned with taxonomies. The weighted UniFrac analysis, a method sensitive to taxa abundances for beta-diversity analysis, expanded dramatically after HFD feeding, especially in the HFD+P group (*R* = 0.85, *P* < 0.01 by ANOSIM test) (Figure 6A). The unweighted UniFrac analysis, which is sensitive to rarer taxa, showed a similarity in the microbiota between HFD and HFD+J group, and a significant shift of HFD+P from other groups was observed (*R* = 0.68, *P* < 0.01 by ANOSIM test) (Figure 6B). Dendrogram of Hierarchical clustering analysis with distance measure based on Bray-Curtis index also confirmed differences between all HFD-fed mice and the CON group (Figure 6C). Alpha diversity analysis of gut microbiota indicated that HFD induced a significant decrease in Shannon index (*P* < 0.05) and in simpson index (*P* < 0.05). No significant difference existed between the two indicators in mice treated with the two bacteria and in mice of the CON or the HFD group (Figure 6D,E). A significant increase was observed in the ratio of Bacteroidetes to Firmicutes in the HFD group compared to that of CON (*P* < 0.05, Figure 6F).

LEfSe analysis showed that *Bacteroidales* S24-7 group and Lachnospiraceae were the marker family of the CON group (Figure 6G,H). The typical marker genus of the HFD group is *Akkermansia* of the Verrucomicrobiaceae family, and this was significantly increased in all mice fed with HFD (*p* < 0.05) (Figure 6F). The HFD+J group was enriched with the genus *Rikenellaceae* RC9 gut group, consisting of an uncultured *Bacteroidales* bacterium and *Coriobacteriaceae*_UCG-002 (Figure 6G). *Alistipes* was the highest marker genus for the HFD+P group (Figure 6G). There was a greater inhibition of the marker genus in the CON group, including *lactobacillus*, than was detected in mice fed a HFD (Table 2).

**Table 2:** Significantly changed genus by comparison using log2 fold change.

	HFD vs. CON		IgA21 vs. CON		HFD+P vs. CON		HFD+J vs. HFD		HFD+P vs. HFD	
	log_2_FC	FDR	log_2_FC	FDR	log_2_FC	FDR	log_2_FC	FDR	log_2_FC	FDR
Eubacterium_coprostanoligenes_group	7.24	< 0.01	5.29	< 0.01					–7.16	< 0.01
Coriobacteriaceae_UCG_002			7.92	< 0.01	6.21	< 0.01	7.16	< 0.01	4.98	< 0.01
Marvinbryantia	–6.20	< 0.01	–6.72	< 0.01	–6.55	< 0.01				
Bacteroides	–1.05	0.04	–1.92	0.03	–1.4	0.03				
Butyricicoccus	–2.89	0.02	–2.18	0.05	–2.13	0.04				
Lachnospiraceae_UCG_001					2.56	< 0.01			2.64	< 0.01
Tyzzerella			–1.94	0.05						
Ruminiclostridium_5	–2.09	0.03	–2.07	0.03	–1.88	< 0.01				
Eubacterium_xylanophilum_group	–1.05	0.04	–3.9	< 0.01	–1.93	< 0.01	–2.63	0.02		
Clostridium_sensu_stricto_1			3.16	0.01						
Family_XIII_UCG_001			2.93	0.01	3.13	< 0.01				
Bifidobacterium									–1.69	0.03
Lactobacillus			–1.94	0.02	–3.02	< 0.01			–2.02	< 0.01
Akkermansia	5.58	< 0.01	4.74	< 0.01	5.03	< 0.01				
Turicibacter	2.22	0.03	4.31	< 0.01	4.03	< 0.01			1.71	0.5
Oscillibacter					1.73	0.02				
Rikenellaceae_RC9_gut_ group	8.76	< 0.01	8.64	< 0.01					–8.78	< 0.01
Ruminococcaceae_UCG_009			2.56	0.01	3.55	< 0.01			1.62	0.05
Ruminococcaceae_UCG_005					2.33	0.03				
Ruminococcaceae_NK4221_group									–1.64	0.047
Erysipelatoclostridium			–2.35	0.02						
Mucispirillum					3.08	< 0.01				
Aerococcus	–3.09	0.04			–3.39	< 0.01				
Staphylococcus					–4.56	< 0.01	2.83	0.04	–3.42	< 0.01
Enterorhabdus					–1.47	0.03			–1.68	0.047
Defluviitaleaceae_UCG_011	3.55	< 0.01	2.61	0.04	3.36	< 0.01				


*Data were expressed as log2 transformed fold change (log_*2*_FC); The differential expression analysis was carried out by edgeR. Only species with FDR ≤ 0.05 and absolute log_*2*_FC ≥ 1.5 are shown; FDR, Adjusted *P*-value.*

At the family level, FS1 treatment induced a significant decrease in the *Bacteroidales* 24-7 group and an increase in *Desulfovibrionaceae* (*P* < 0.05 vs. CON) (Figure 6F). At the genus level (Table 2), IgA21 significantly increased the Eubacterium coprostanoligenes group, the Clostridium sensu stricto 19 group, and the Rikenellaceae RC9 gut group while causing a decrease in *Tyzzerella* and *Erysipelatoclostridium* compared to levels detected in CON (FDR < 0.01). FS1 mainly promoted the proliferation of *Lachnospiraceae* UCG 001, *Oscillibacter*, *Ruminococcaceae* UCG 005, and *Mucispirillum*, but it inhibited *Staphylococcus* and *Enterorhabdus* (FDR < 0.01).

## Discussion

This study confirmed that *Lactobacillus* was the commonly found genus of IgA-coated bacteria in human feces, which is in agreement with other studies (Bjursell et al., 2008). *L. jensenii* is the main species that we isolated from a healthy woman. This species was previously found to be dominant in the vaginal microbiota of healthy women (Pavlova et al., 2002), and it is known to show a strong ability to form biofilms (Ventolini et al., 2015). It is, however, rarely encountered in human feces (Rossi et al., 2016) and almost disappears in individuals with IgA defects (Lonnermark et al., 2012). Compared to the specific lumen lactic acid bacteria FS1, IgA21 exhibits stronger surface hydrophobicity, mucus adhesion, and a higher growth rate, suggesting that it may be more adaptable to a mucus environment. This demonstrates that IgA -coated bacteria may live near epithelial cells in the mucous layer of the human or mammalian intestine (Pabst et al., 2016).

In the following short term HFD feeding experiment, mice showed significant weight gain without development of obesity (weight gain less than 20%). We found that HFD induced a significant increase in ambulatory activity. This is in agreement with a study by Kohsaka et al. (2007) who found an increase in the free-running period in mice within 6 weeks on a HFD. Although the two bacteria significantly reduced the ambulatory activity of mice fed with HFD, they did not significantly increase body weight, suggesting that the ambulatory activity of mice was independent of body weight.

Probiotics may prevent the occurrence of HFD-induced dyslipidemia by regulating intestinal flora. (Aron-Wisnewsky et al., 2013). In the present study, it was demonstrated that IgA21, but not FS1, significantly lowered hypertriglyceridemia and hypercholesterolemia in HFD-fed mice. The results also confirmed alteration of gut barrier in HFD-fed mice following endotoxemia and low-grade inflammation marked by increased serum TNF-α, and treatment with IgA21 restored the damaged barrier. This low-grade inflammation alters lipid metabolism (Cani et al., 2007). It was found that TNF-α can suppress lipoprotein lipase synthesis, contributing to hypertriglyceridemia due to decrease of peripheral clearance rate of triglyceride (Kawakami et al., 1987). *De novo* fatty acid synthesis would be stimulated by low doses of LPS in the liver in conjunction with increased lipolysis, providing more fatty acids for hepatic TG production (Feingold et al., 1992). The role of IgA21 in alleviating lipid disorders may be related to its enhancement of the mucosal barrier, which can inhibit endotoxemia and chronic inflammation. Intestinal barrier is the result of interaction between host and microorganism, including physical barrier, immune barrier, and microbial colonization resistance. In this study, IgA21 may regulate the mucosal barrier in three ways. First, it upregulated the expression of mucin 2, which is important for the formation of the mucous layer that separates epithelial cells from harmful antigens and is an important chemical barrier to prevent pathogenic bacterial infection (Wagner et al., 2018). It has been found that a HFD decreases gut goblet cell expression of mucin-2 associated with reduced expression of Zonula occludens-1 and Occludin mRNA (Lee et al., 2017). Second, PIgR is a gene related to mucus secretion of sIgA (Johansen and Kaetzel, 2011), which is a major target of IgA21. IgA secreted within the gut plays an important role in maintaining gut immunological barrier function by inhibiting pathogens from adhering to the mucous, thus exerting effects in the lumen [29]. Third, IgA21 elicits anti-inflammatory activity within the intestine that is marked by decreased TNF-alpha production and increased activity of IAP. Other *L. jensenii* strains such as TL2937 were also found to mediate the induction of negative regulators of TLRs and prevent intestinal inflammatory damage (Shimazu et al., 2012). TNF-α is a key cytokine that can lead to mucosal barrier damage by inducing cytoskeleton depolymerization (Gibson, 2004). Last, IgA21 promotes the production of butyrate to enhance gut barrier function (Peng et al., 2009). Up-regulation of Claudin-2 can potentially affect the structure and function of tight junctions, resulting in barrier dysfunction (Wang et al., 2017). IgA21 administration also prevented HFD induced over-expression of Claudin-2, which may be related to stimulation of butyrate production. It has found that butyrate represses claudin-2 mRNA expression, a gene related to permeability-promoting tight-junction proteins (Zheng et al., 2017), through an IL-10 receptor A-dependent mechanism (Zheng et al., 2017).

This study also demonstrated that short-term HFD caused significant changes in the structure of intestinal microbiota, and FS1 treatment caused further disturbance, while IgA21 did not exert a significant impact according to beta diversity. The mucus digesting bacteria *Akkermansia* was the typical marker bacterium that was increased by HFD. The impact of HFD on *Akkermansia* is controversial, where some studies have demonstrated that a HFD reduced gut *Akkermansia* (Everard et al., 2013), while other studies demonstrated the opposite result (Carmody et al., 2015). Recently, it has also been found that mice receiving a high-fat, ketogenic diet for 2 weeks exhibited a significant increase in gut *Akkermansia* (Olson et al., 2018), which suggested that mucus of mice fed a high-fat diet became the main carbon source to support their growth. The main difference between FS1 and IgA21 is that the former significantly inhibits the proliferation of the *Bacteroidales* S24-7 group and promotes the proliferation of *Desulfovibrionaceae* at the family level (Figure 6H). S24-7, an IgA-targeted gut commensal in humans and mouse, is involved in carbohydrate fermentation and utilization (Palm et al., 2014; Ormerod et al., 2016). *Desulfovibrionaceae* are sulfate-reducing and endotoxin-producing bacteria that were highly enriched in mice by long term high-fat feeding (6 months) and were associated with the development of metabolic syndromes (Zhang et al., 2010). They play a role in reducing sulfate to H_2_S to damage the gut barrier. These changes in the two types of bacteria indicated that FS1 promoted intestinal bacterial imbalance induced by HFD.

At the genus level, IgA21 is associated with enriched *Eubacterium coprostanoligenes* group, a typical cholesterol-reducing bacterium (Li et al., 1998), which may explain to some extent its ability to prevent hyperlipidemia. *Mucispirillum* is a genus specially enriched by FS1treatment, and this bacteria is a pathobiont within the mucus layer of the rodent gut which is associated with inflammation (Loy et al., 2017). This may be partially related to the failure of FS1 to maintain the mucosal barrier effectively.

## Conclusion

In conclusion, IgA-coated and non-coated lactic acid bacteria within the gut have been shown to differentially affect the intestinal barrier and serum lipids. This indicates that IgA-bound bacteria possess the potential to more easily interact with the host gut to regulate homeostasis.

## Data Availability

The raw data were uploaded to SRA database and the BioProject ID is PRJNA523678.

## Ethics Statement

IgA-coated bacteria were enriched and cultured from samples of 12 healthy female provided by the affiliated Changzhou Maternity and Child Health Care Hospital of Nanjing Medical University according to a protocol approved by the Internal Ethics Committee of the Institute of Chinese Medical Sciences, Jiangnan University. Written informed consent was obtained from all donors. This animal experiments was performed according to the National Guidelines for Experimental Animal Welfare (MOST of PR China, 2006), and approved by the Jiangnan University Animal Ethics Committee approved all experiments under protocol number 128/16.

## Author Contributions

CQ and RY participated in data collection, performed the analyses, and wrote the manuscript. HZ designed the study, collected the data, and participated in data analysis. QZ, DC, HX, RY, and GL participated in data analysis and writing of the manuscript. HX, GL, JS, and HZ performed data preprocessing. JS participated in study design and writing of the manuscript and was responsible for overall study coordination.

## Conflict of Interest Statement

The authors declare that the research was conducted in the absence of any commercial or financial relationships that could be construed as a potential conflict of interest.

## Footnotes

1 http://ccb.jhu.edu/software/FLASH/
