# Supplementary material for: IgA-Targeted Lactobacillus jensenii Modulated Gut Barrier and Microbiota in High-Fat Diet-Fed Mice
Source: Front Microbiol. 2019 May 24;10:1179. doi: 10.3389/fmicb.2019.01179 (PMC6542990; doi:10.3389/fmicb.2019.01179)
Supplement: Supplementary file 1 [file Data_Sheet_1.pdf]

Supplementary Material

1. Supplementary Table

Supplementary Table S1. Basic physical characteristics and blood lipids profile of volunteers

	Population	Standard range
Age (years)	23.45(3.24)	-
Body mass index (kg m^{-2})	23.03(2.55)	18~24.9
Waist hip ratio	0.74(0.05)	0.67~0.80
Waist circumserence (cm)	71.58(12.33)	>80
Hip circumference (cm)	96.72(15.47)	>90
HDL-Cholesterol (mg/dl)	66.08(16.35)	33~77
LDL-Cholesterol (mg/dl)	87.49(28.58)	80~120
Triglycerides (mg/dl)	82.74(42.96)	>150

Values are given as means and standard deviations (SD)

Supplementary Table S2. PCR primers used in AFLP and mRNA expression analysis

Name	Sequence	Annealing temperature (^oC)
Bacteria identification		
27F	AGAGTTTGATCMTGGCTCAG	56
2792R	TACGGYTACCTTGTTACGACTT	
AFLP analysis		
EcoR I adapter	F:CTCGTAGACTGCGTACC	65
	R:AATTGGTACGCAGTC	
Mse I adapter	F:TACTCAGGACTCAT	65
	R:GAGTCCTGAGTAGCAG	
EcoRI core	F:GACTGCGTACCAATTC	56
MseI core	R:GATGAGTCCTGAGTAA	
EcoRI-A	F:GACTGCATACCACCAATTCA	55
MseI-CA	R: AATGAGTCCTGAGTAGCA	
mRNA expression		
β-action	F: TGACGTTGACATCCGTAAAGACC	60
	R: CTCAGGAGGAGCAATGATCTTGA	
ZO-1	F: TACCTCTTGAGCCTTGAACTT	60
	R: CGTGCTGATGTGCCATAATA	
Occludin	F: GTGTGGTTGATCCCCAGGAG	60
	R: TCGCTTGCCATTCACTTTGC	
Claudin-2	F: CCCAGGCCATGATGGTGA	60
	R: TCATGCCCACCACAGAGATAAT	
pIgR	F: AGTAACCGAGGCCTGTCCTT	60
	R: GTCACTCGGCAACTCAGGA	
Mucin-2	F: CCCAGAAGGGACTGTGTATG	59
	R: TGCAGACACACTGCTCACA	

AFLP: Amplified fragment length polymorphism; pIgR, The polymeric immunoglobulin receptor; ZO-1, Zonula occludens-1

Supplementary Table S3. Animal feed formulation

Ingredient	Proportion (%)	
	Normal control	High fat diet
Corn flour	48.38	30.3
Wheat flour	9	9
Wheat bran	9	9
Soybean meal	24.52	24.52
Soybean oil	2.8	2.8
Lard	2.54	20.62
Sucrose	0.1	0.1
Lysine	0.28	0.28
Methionine	0.2	0.2
CaHPO3	1.2	1.2
CaCO3	1.6	1.6
NaCl	0.2	0.2
Choline chloride	0.1	0.1
Mixed vitamin[a]	0.02	0.02
Mixed minerals[b]	0.06	0.06

[a] Mixed vitamin (per 1 Kg): Vitamin A 1×10^6 IU, Vitamin D3 5×10^4 IU, Vitamin E 5×10^2 IU, Vitamin K_3 100 mg， Vitamin B_1 100 mg, Vitamin B_2 100 mg, Vitamin B_6 200 mg, Vitamin B_{12} 5 mg, niacin 100 mg, thbrthdrexvbdr 200 mg, Add sucrose to 1 Kg; [b]Mixed minerals (per 1 Kg): $FeSO_4{\bullet}H_2O$ 255 g, $GuSO_4{\bullet}H_2O$ 27.9 g, $MnSO_4{\bullet}H_2O$ 253.8 g, $ZnSO_4{\bullet}H_2O$ 137 g, Na_2SeO_3 0.5 g, KI 0.7 g, Add sucrose to 1 Kg.

Supplementary Table S4 Identification of strains

AFLP pattern	Number	Reference strain	Similarity (%)
A1	3	*Lactobacillus jensenii* DSM 20557	96.5
A2	16	*Lactobacillus jensenii* FN1621	99
A3	4	*Lactobacillus jensenii* DSM 20557	100
A4	5	*Lactobacillus jensenii* DSM 20557	99
B1	5	*Lactobacillus jensenii* FN1621	99
B2	6	*Lactobacillus jensenii* FN1621	99
C1	1	*Lactobacillus jensenii* FN1621	99
Ⅰ	8	*Pediococcus acidilactici* NGRI 0510Q	99
ⅠⅠ	7	*Pediococcus acidilactici* NGRI 0510Q	99.9
ⅠⅠⅠ	1	*Pediococcus acidilactici* FN1701	99.8

AFLP: Amplified fragment length polymorphism

2. Supplementary Figures

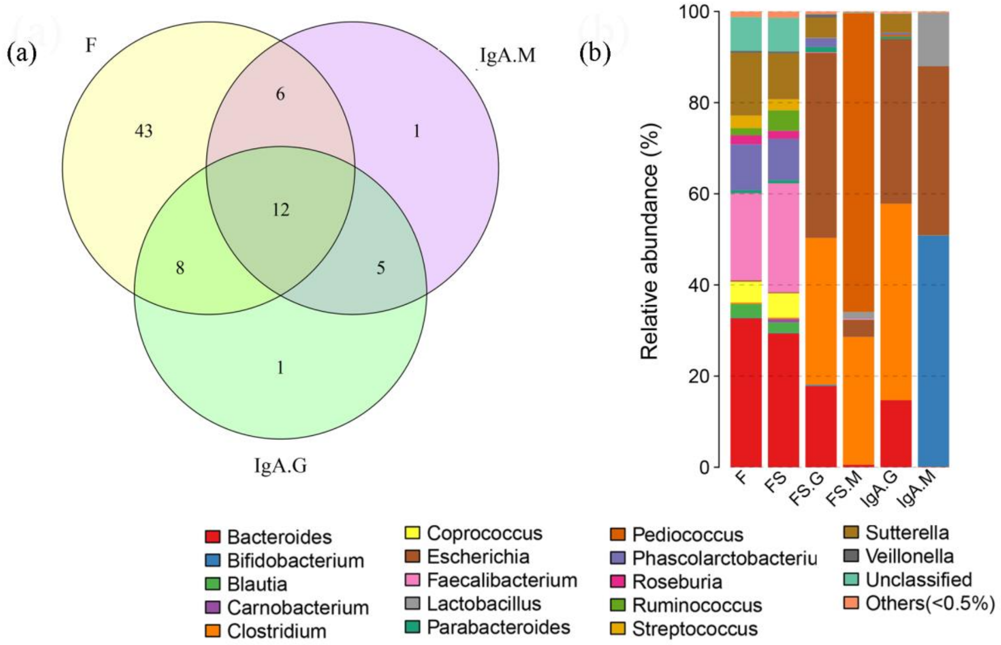

Supplementary Figure S1. Community structure of gut microbiota medium (GMM) and De Man Rogosa Sharpe (MRS) cultured IgA coated bacteria from a healthy female donor. (a) Venn diagrams comparing the OTU memberships of feces microbiota (F), GMM (IgA.G) and MRS (IgA.M) cultured IgA coated microbiota. (b) Genus composition of feces microbiota (F), IgA free microbiota (FS), and GMM and MRS cultured IgA coated microbiota and IgA free microbiota (FS.G and FS.M).

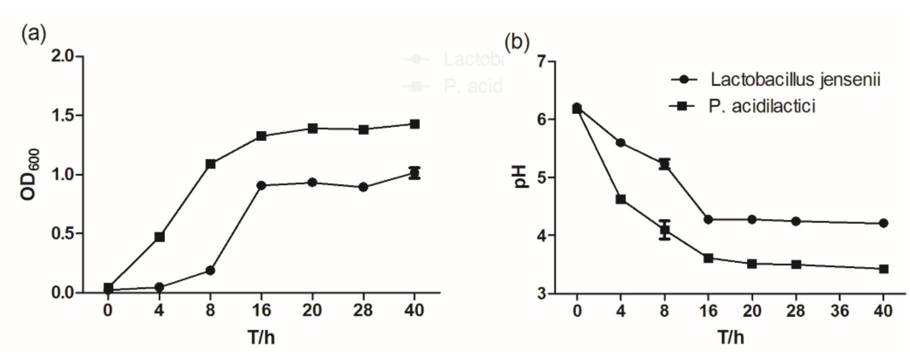

Supplementary Figure S2. Mean growth curve (a) and pH change (b) during fermentation of *Lactobacillus jensenii* IgA21 (n=6) and *P. acidilactici* FS1 (n=6).

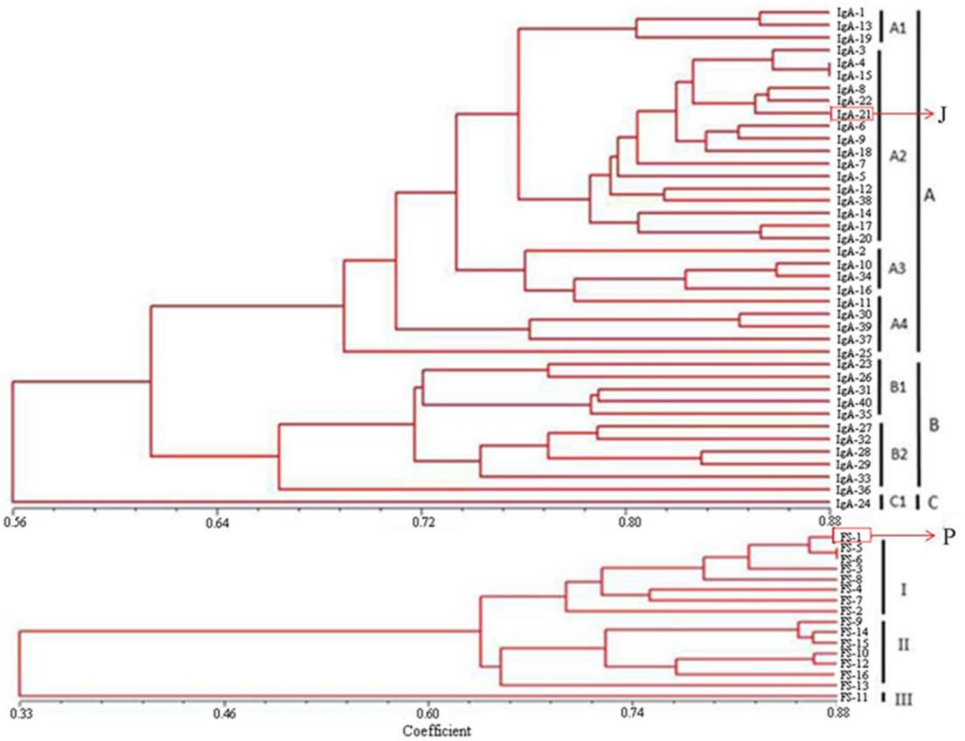

Supplementary Figure S3. Dendrogram generated using the unweighted pairgroup method with arithmetic averages (UPGMA) cluster analysis, with Dice's coefficient based on the amplified fragment length polymorphism (AFLP) profiling of the *Lactobacillus jensenii* and *Pediococcus acidilactici* strains isolated from IgA positive and free microbiota of human feces.

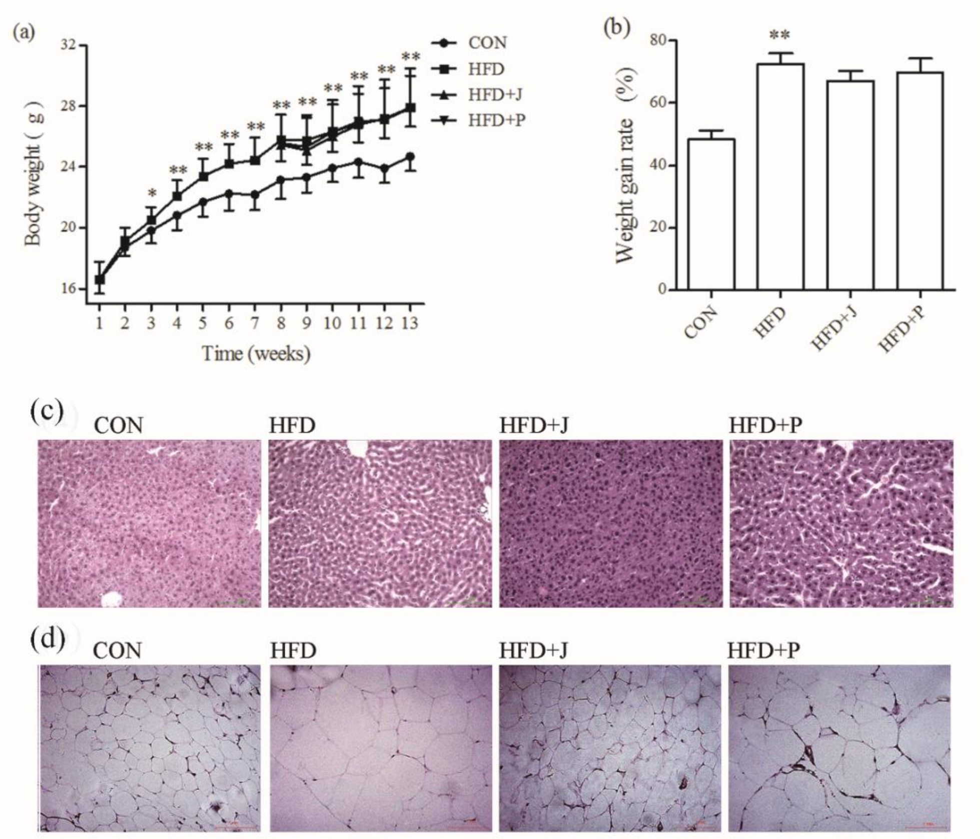

Supplementary Figure S4. Body weight change (a), weight gain rate (b), and hematoxylin and eosin (H&E) stains of liver (c) and white fat tissue (d). ** $P<0.01$ compared with CON. CON, control group; HFD, high fat diet group; HFD+J, HFD mice treated with *Lactobacillus jensenii* IgA21; HFD+P, HFD mice treated with *Pediococcus acidilactici* FS1
